# Comparative assessment of *An. gambiae* and *An. stephensi* mosquitoes to determine transmission-reducing activity of antibodies against *P. falciparum* sexual stage antigens

**DOI:** 10.1186/s13071-017-2414-z

**Published:** 2017-10-17

**Authors:** Maarten Eldering, Anaïs Bompard, Kazutoyo Miura, Will Stone, Isabelle Morlais, Anna Cohuet, Geert-Jan van Gemert, Patrick M. Brock, Sanna R. Rijpma, Marga van de Vegte-Bolmer, Wouter Graumans, Rianne Siebelink-Stoter, Dari F. Da, Carole A. Long, Merribeth J. Morin, Robert W. Sauerwein, Thomas S. Churcher, Teun Bousema

**Affiliations:** 1 Department of Medical Microbiology, Radboud University Medical Center, Nijmegen, The Netherlands; 2 MRC Centre for Outbreak Analysis and Modelling, Department of Infectious Disease Epidemiology, Imperial College London, London, UK; 3 National Institute of Allergy and Infectious Diseases, Laboratory of Malaria and Vector Research, National Institutes of Health, Rockville, MD USA; 4 Institut de Recherche pour le Développement, UMR MIVEGEC UM-CNRS 5290-IRD 224, Montpellier, France; 5 Institut de Recherche en Sciences de la Santé, Bobo Dioulasso, Burkina Faso; 6 Institute of Biodiversity, Animal Health and Comparative Medicine, College of Medical, Veterinary and Life Sciences, University of Glasgow, Glasgow, UK; 7 PATH Malaria Vaccine Initiative, Washington DC, USA; 8 Department of Immunology and Infection, London School of Hygiene and Tropical Medicine, London, UK

**Keywords:** Malaria, *Anopheles*, Transmission, Vaccine, Immunity

## Abstract

**Background:**

With the increasing interest in vaccines to interrupt malaria transmission, there is a demand for harmonization of current methods to assess *Plasmodium* transmission in laboratory settings. Potential vaccine candidates are currently tested in the standard membrane feeding assay (SMFA) that commonly relies on *Anopheles stephensi* mosquitoes. Other mosquito species including *Anopheles gambiae* are the dominant malaria vectors for *Plasmodium falciparum* in sub-Saharan Africa.

**Methods:**

Using human serum and monoclonal pre-fertilization (anti-Pfs48/45) and post-fertilization (anti-Pfs25) antibodies known to effectively inhibit sporogony, we directly compared SMFA based estimates of transmission-reducing activity (TRA) for *An. stephensi* and *An. gambiae* mosquitoes.

**Results:**

In the absence of transmission-reducing antibodies, average numbers of oocysts were similar between *An. gambiae* and *An. stephensi*. Antibody-mediated TRA was strongly correlated between both mosquito species, and absolute TRA estimates for pre-fertilisation monoclonal antibodies (mAb) showed no significant difference between the two species. TRA estimates for IgG of naturally exposed individuals and partially effective concentrations of anti-Pfs25 mAb were higher for *An. stephensi* than for *An. gambiae*.

**Conclusion:**

Our findings support the use of *An. stephensi* in the SMFA for target prioritization. As a vaccine moves through product development, better estimates of TRA and transmission-blocking activity (TBA) may need to be obtained in epidemiologically relevant parasite-species combination.

**Electronic supplementary material:**

The online version of this article (10.1186/s13071-017-2414-z) contains supplementary material, which is available to authorized users.

## Background

Recent declines in *Plasmodium falciparum* malaria transmission intensity in several African settings, associated with the wide-scale deployment of efficacious vector control and artemisinin combination therapy [1], have contributed to a renewed interest in the elimination of malaria. Further scaling up of control efforts with currently available tools is unlikely to achieve this goal in most African settings [2] and interventions that specifically target malaria transmission are considered highly desirable to accelerate elimination efforts [3]. Vaccines that interrupt malaria transmission (VIMT) are high on the priority list for malaria elimination [4]. These include classical transmission-blocking vaccines that target sexual, sporogonic, or mosquito stages of the parasite (SSM-VIMT) and interfere with parasite transmission in the vector host. Recent success in obtaining properly folded vaccine candidates that elicit functional transmission-blocking immunity in animal models have paved the way for clinical development of lead SSM-VIMT candidates (reviewed in [5–7]). For *P. falciparum*, these candidates include vaccines based on parasite antigens Pfs230, Pfs48/45, Pfs25 and mosquito antigen AnAPN1 [5] acting on different life-stages during sporogonic development.

Malaria transmission starts with the ingestion of male and female gametocytes by blood-feeding anophelines. Upon ingestion, gametocytes quickly differentiate into male microgametes and female macrogametes that fuse to form zygotes. Within 18–24 h this zygote turns into a motile ookinete which penetrates the mosquito midgut epithelium and differentiates into an oocyst [8]. Over the next two weeks sporozoites do develop inside the oocyst, eventually bursting the oocyst capsule before migrating to and invading the salivary glands, rendering the mosquito infective to humans. Both vaccine-induced and naturally acquired antibodies against Pfs230 and Pfs48/45 can prevent fertilization and thus zygote formation [9–12]. Antibodies against Pfs25 prevent ookinete penetration of the mosquito midgut [13, 14] while antibodies against AnAPN1 also block ookinete penetration by preventing the parasite interacting with mosquito midgut ligands [15, 16].

Clinical development of SSM-VIMTs has been primarily guided by assessments of TRA in the SMFA that utilizes the African *P. falciparum* strain NF54 and *Anopheles stephensi* mosquitoes [17, 18]. The SMFA also forms the gold standard assay to assess naturally acquired transmission-reducing immunity [18]. *Anopheles stephensi* is a primary malaria vector in the Indian subcontinent but not in Africa where *A. funestus* and *An. gambiae* (*s.l.*) dominate [19]. *Anopheles stephensi* and *An. gambiae* mosquitoes belong to the subgenus *Cellia*, a group of major Old World malaria vectors with a wide geographical distribution that diversified millions of years ago [20]. There are several factors in mosquito physiology and immunity that may contribute to differences in the vectorial competence of *An. stephensi* and *An. gambiae* [21–24]. These potential differences raise questions about the extent to which SMFA results, commonly using *An. stephensi*, can be directly translated to *An. gambiae.* Here, we compared *An. stephensi* and *An. gambiae* in terms of transmission efficiency and TRA estimates in the SMFA using transmission-blocking mAb and sera of naturally gametocyte-exposed individuals.

## Methods

### Mosquito rearing, parasite culture and standard membrane feeding assay

*Anopheles stephensi* (Sind-Kasur Nijmegen strain) [25] and *An. gambiae* (*s.s.*) (Ngousso strain) [26] were reared at 30 °C and 70–80% humidity, and exposed to a 12/12 h day/night cycle. Mature *P. falciparum* (NF54) gametocytes (14 day culture, 0.3–0.5% gametocytes, 2% haematocrit) were obtained from an automated tipper system and prepared as previously described [27, 28]. For infection experiments, 3–5 day-old mosquitoes were fed on a glass membrane feeder system containing 270 μl of *P. falciparum* culture mixture [28]. Unfed and partially fed mosquitoes were removed from the samples. Blood-fed mosquitoes were maintained at 26 °C and 70–80% humidity. Routine dissection and staining of midguts was done 6–9 days post-infection in 1% mercurochrome solution for oocysts quantification.

### Antibody preparations

#### Monoclonal antibodies

Four monoclonal antibodies against two *P. falciparum* antigens were selected for the SMFA species comparison. Rat mAb 85RF45.1 and 85RF45.5, against pre-fertilization protein Pfs48/45 epitopes 1 and 5, respectively [29] and rat mAb 32F81 [30] and mouse mAb 4B7 against [31] post-fertilization protein Pfs25. Concentrations of mAb were chosen to achieve full and partial TRA in *An. stephensi.* All mAb were diluted in human serum aiming for a final concentration in the feeder for Pfs48/45-85RF45.1: 10, 2.5, 0.63 and 0.16 μg/ml; Pfs48/45-85RF45.5: 60, 20, 6.7 and 2.2 μg/ml; Pfs25-4B7: 94, 23.5, 5.9 and 1.47 μg/ml; Pfs25-32F81: 15, 10, 5, 2.5, 1.67, 1.25, 0.63 and 0.56 μg/ml. Human serum was used as a control for TRA assessments. In the models that assessed the association between antibody concentration and TRA, we also included other experiments conducted with the same mAb in the same lab in the period 1998–2015 with additional antibody concentrations for Pfs48/45-85RF45.1 (20–0.04 μg/ml), Pfs48/45-85RF45.5 (100–6.25 μg/ml) and Pfs25-32F81 (20–0.63 μg/ml).

#### Human test sera

Two sets of plasma samples were selected for analysis in the SMFA species comparison. One set (*n* = 5) comprised plasma samples from European expatriates who lived in malaria endemic regions for more than 10 years, and showed strong and consistent TRA in the SMFA [18, 32]. A second set (*n* = 21) comprised plasma samples derived from gametocyte carriers living in endemic regions in Cameroon (Yaoundé, 2010–2012) and Burkina Faso (Bobo-Dioulasso, 2012). Samples were collected for ex vivo infectivity assessments as reported previously [33]. Ethical approval was obtained from the Centre Muraz Institutional Ethics Committee under agreement number 0003–2009/CE-CM. The protocol conforms to the declaration of Helsinki on ethical principles for medical research involving human subjects (version 2002) and informed written consent were obtained from all volunteers.

IgG was purified from 300 μl plasma samples using Protein G HP Spintrap (GE Healthcare, Chicago, IL, US) according to the manufacturer’s instructions and as previously described [18, 34] and reconstituted in 300 μl of milliQ water. Yield was 70–80% and IgG concentrations were 7–18 mg/ml measured with the NanoDrop ND-1000 (Thermo Scientific, Waltham, MD, US). IgG was tested by mixing 90 μl of plasma IgG in freeze-dried foetal calf serum (FCS) (original volume was 90 μl) and added to 180 μl of gametocyte/red blood cell mix (containing 30 μl of active complement containing serum) to a total volume of 270 μl. Primary control samples were 90 μl of freeze-dried FCS dissolved in 90 μl milliQ water. Subsidiary controls (IgG without TRA from an expatriate and individuals from Cameroon and Burkina Faso) were also tested. In addition, 30 μl of active complement containing human serum (Sanquin Blood Supply, Nijmegen, Netherlands) was included in the culture mix for all serum IgG experiments. No significant differences were observed between IgG and FCS controls. Human IgG controls and FCS were used as the primary controls as they were available for all experiments. All SMFAs were blinded for evaluation and analysis.

### Statistical analysis

For our analysis we only included experiments with a minimum of 20 dissected mosquitoes per group and a minimum infection prevalence of 70% in the control serum/IgG group (excluded experiments available in Additional file 1: Table S1) [32]. Statistical analyses were performed using R software (v 3.2 [35])) and R package *glmmADMB* (v 11.6, [36]). TRA was estimated as the relative reduction in oocyst intensity in the treatment relative to the control. First, Spearman's correlation tests were performed on TRA results for both species to determine whether use of one species over another would influence candidate prioritization. Subsequently, generalized linear mixed models (GLMMs) were used to assess the potential species effect on absolute TRA, assuming a zero-inflated negative binomial distribution for oocysts counts. For each antibody type, treatment, antibody concentration and mosquito species were tested as fixed effects whilst replicate feeds were included as a random effect to correct for variance between feedings [37]. Estimates of TBA as the relative reduction in oocyst prevalence (using GLMMs with a binomial distribution) are presented in Additional file 1: Table S1, Additional file 2: Figure S1, Additional file 3: Figure S2 and Additional file 4: Figure S3. Intervention TBA has been shown to vary according to the level of parasitemia in the mosquito population (as assessed by mean oocyst intensity in control mosquitoes) [37, 38]. Since the overall number of oocysts might vary between mosquito species, TRA is used for the comparison between mosquito species in the main text. All statistics were carried out on the complete dataset though the figures and TRA estimates were obtained by using independent GLMMs for each individual antibody. To aid visual clarity, experiments where oocyst intensities were higher in test mosquitoes than controls were given an efficacy of zero in the figures but were left unaltered in the statistical analyses.

## Results

**Fig. 1:**
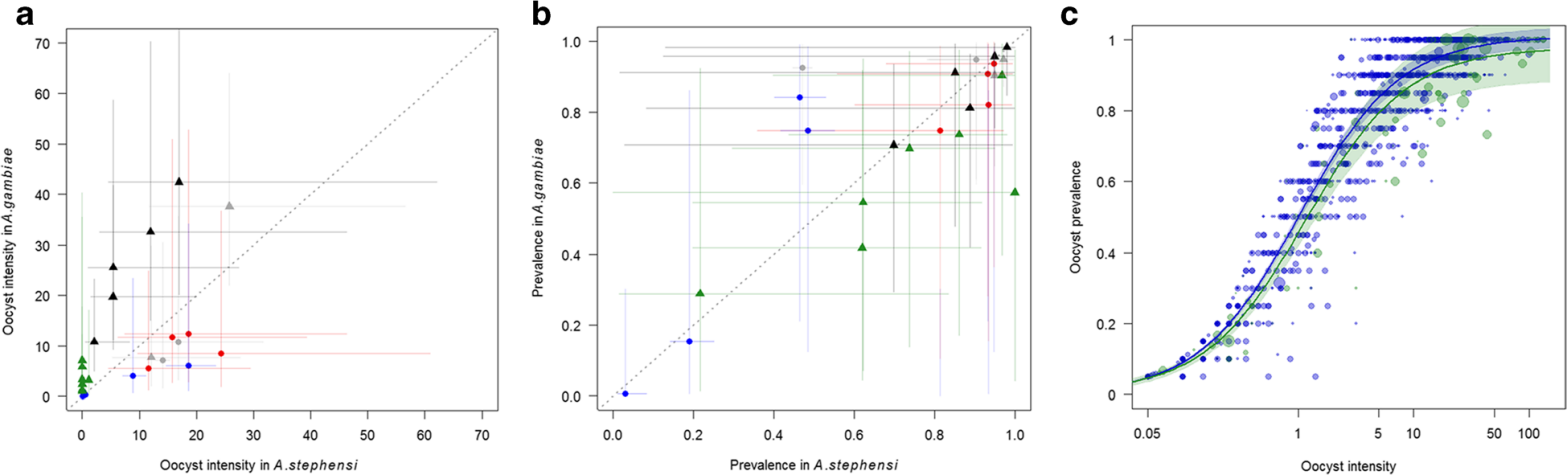
Prevalence and oocyst intensity in *An. stephensi* and *An. gambiae* mosquitoes. **a** Mean oocysts intensity (dots) with 95% confidence intervals (lines) in *An. gambiae* depending on mean oocyst intensity with 95% confidence intervals for *An. stephensi*, for control groups (*grey*), mAb Pfs48/45-85RF45.1 (*blue*), mAb Pfs48/45-85RF45.5 (*red*), mAb Pfs25-32F81 (*green*) and mAb Pfs25-4B7 (*black*). **b** Mean prevalence in oocysts (dots) with 95% confidence intervals (lines) in *An. gambiae* depending on mean prevalence with 95% confidence intervals for *An. stephensi*. Same color codes as for panel **a**. **c** Relationship between oocysts prevalence and intensity for *An. stephensi* (*blue*) and *An. gambiae* (*green*). The line and shaded area represent the predicted relationship and predicted confidence interval. Each data point represents the mean oocyst intensity and oocyst prevalence in a single group of mosquitoes. The size of each data point represents the size of the mosquito group analyzed. Data were collected from both control and experimental feeds between 6 and 9 days post infection. In total 1635 separate feedings comprising 25,574 mosquito dissections are shown: 150 feeds with *An. gambiae* (*green*) comprising 2691 mosquitoes and 1485 feeds with *An. stephensi* (*blue*) comprising 22,883 mosquitoes

The mean oocyst intensity and the prevalence of mosquitoes infected with oocysts in *An. gambiae* and *An. stephensi* that were fed the same blood meal without any interventions were directly compared (Fig. 1a, b). Mean oocyst intensities in control feeds ranged from 2.9 to 113.7 in *An. stephensi* (oocyst prevalence 70–100%) and 4.4 to 80.1 for *An. gambiae* (oocyst prevalence 80–100%). There was a broad correlation across the whole dataset between oocyst intensities in *An. gambiae* and *An. stephensi* (Spearman's correlation coefficient *r* = 0.92, 95% confidence interval (CI): 0.71–0.98, *P* < 0.0001). Absolute average numbers of oocysts were not significantly different between *An. gambiae* and *An. stephensi* in control groups; 25.32 (95% CI: 15.13–34.27) oocysts/mosquitoes for *An. stephensi* vs 22.77 (95% CI: 19.67–32.58) oocysts/mosquitoes for *An. gambiae* (GLM, *z*_(nb. of observations = 2194)_ = 1.13, *P* = 0.18. The absolute prevalence of oocysts in *An. stephensi* (83.79%; 95% CI: 59.27–83.81) was statistically significantly higher than in *An. gambiae* (73.26%; 95% CI: 64.33–93.84; *z*_(2194)_ = 2.55, *P* = 0.01).

The shape of the relationship between oocyst prevalence and oocyst intensity was tested in a larger dataset where the two mosquito species were not fed on the same blood sources. This comprises all SMFA experiments conducted between January 2011 and January 2013. The shape of the relationship between prevalence and intensity was similar for both species (Fig. 1c), suggesting the distribution of oocysts across mosquitoes is the same within *An. gambiae* and *An. stephensi*.

### Assessments of transmission-reducing activity of monoclonal antibodies

**Fig. 2:**
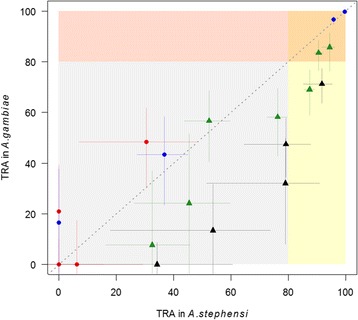
The ranking of estimates of TRA of antibodies against Pfs48/45 (mAb 85RF45.1 and mAb 85RF45.5) and Pfs25 (mAb 32F81 and mAb 4B7) in *An. gambiae* and *An. stephensi* mosquitoes. TRA of transmission effective mAb 85RF45.1 (blue), 85RF45.5 (red), 32F81 (green) and 4B7 (black) in *An. gambiae* depending on TRA in *An. stephensi* mosquitoes. Dots and triangles represent the predicted TRA, while lines represent 95% confidence intervals in *An. gambiae* and *An. stephensi*

TRA of four monoclonal antibodies, targeting Pfs48/45 (mAb 85RF45.1 and mAb 85RF45.5) and Pfs25 (mAb 32F81 and mAb 4B7) was investigated using *An. stephensi* and *An. gambiae.* In experiments performed in both species, there was a very strong correlation between SMFA estimates using *An. stephensi* and *An. gambiae* (Spearman's correlation coefficient *r* = 0.80, 95% CI: 0.65–0.89%, P < 0.0001). If experiments were ranked in order of magnitude of TRA, the two species did not rank all experiments in the same order. Six mAb concentrations combinations resulted in > 80% TRA in *An. stephensi* (mAb 85RF45.1 at 10 and 2.5 μg/ml; mAb 32F81 at 15, 10 and 5 μg/ml; mAb 4B7 at 94 μg/ml); 4 of these also showed > 80% TRA in *An. gambiae* (mAb 85RF45.1 at 10 and 2.5 μg/ml; mAb 32F81 at 15 and 10 μg/ml) (Fig. 2). All highly active mAb concentrations tested in our laboratory colony of *An. gambiae* also showed high efficacy in *An. stephensi*. For anti-Pfs25 antibody concentrations with intermediate TRA, the efficacy was sometimes slightly overestimated in *An. stephensi*. Similar patterns were seen between species for transmission-blocking activity (TBA), showing no significant difference for all mAbs (mAb 85RF45.1, *z*_(2133)_ = 0.67, *P* = 0.50; mAb 85RF45.5, *z*_(2969)_ = -0.45, *P* = 0.65; mAb 4B7, *z*_(1070)_ = -0.93, *P* = 0.35; mAb 32F81, *z*_(2594)_ = -1.66 *P* = 0.096) (Additional files 2: Figure S1, Additional file 3: Figure S2). However, variance in TBA estimates was greater than TRA estimates since TBA is heavily dependent on control oocysts intensity [37, 38]. Control oocyst intensities vary considerably between experiments and, although this does not affect the validity of TRA estimates, high control oocyst intensities frequently lead to situations where oocyst prevalence is 100% and a considerable reduction in oocyst density is required before oocyst prevalence is affected, making TBA as outcome measure less relevant. For our experimental approach, TRA estimates were therefore considered more robust and more relevant than TBA estimates.

Although TRA outcomes of different mAb concentrations were ranked in broadly the same order, the actual TRA estimates showed some differences between *An. gambiae* and *An. stephensi*, demonstrated by a significant interaction term between intervention and mosquito species using GLMM analysis (*z*_(8766)_ = -2.71, *P* = 0.0067). The difference between mosquito species varied between antibody type and the efficacy of the intervention. To illustrate this, the impact of varying titers on TRA was assessed separately for the different antibodies.

**Fig. 3:**
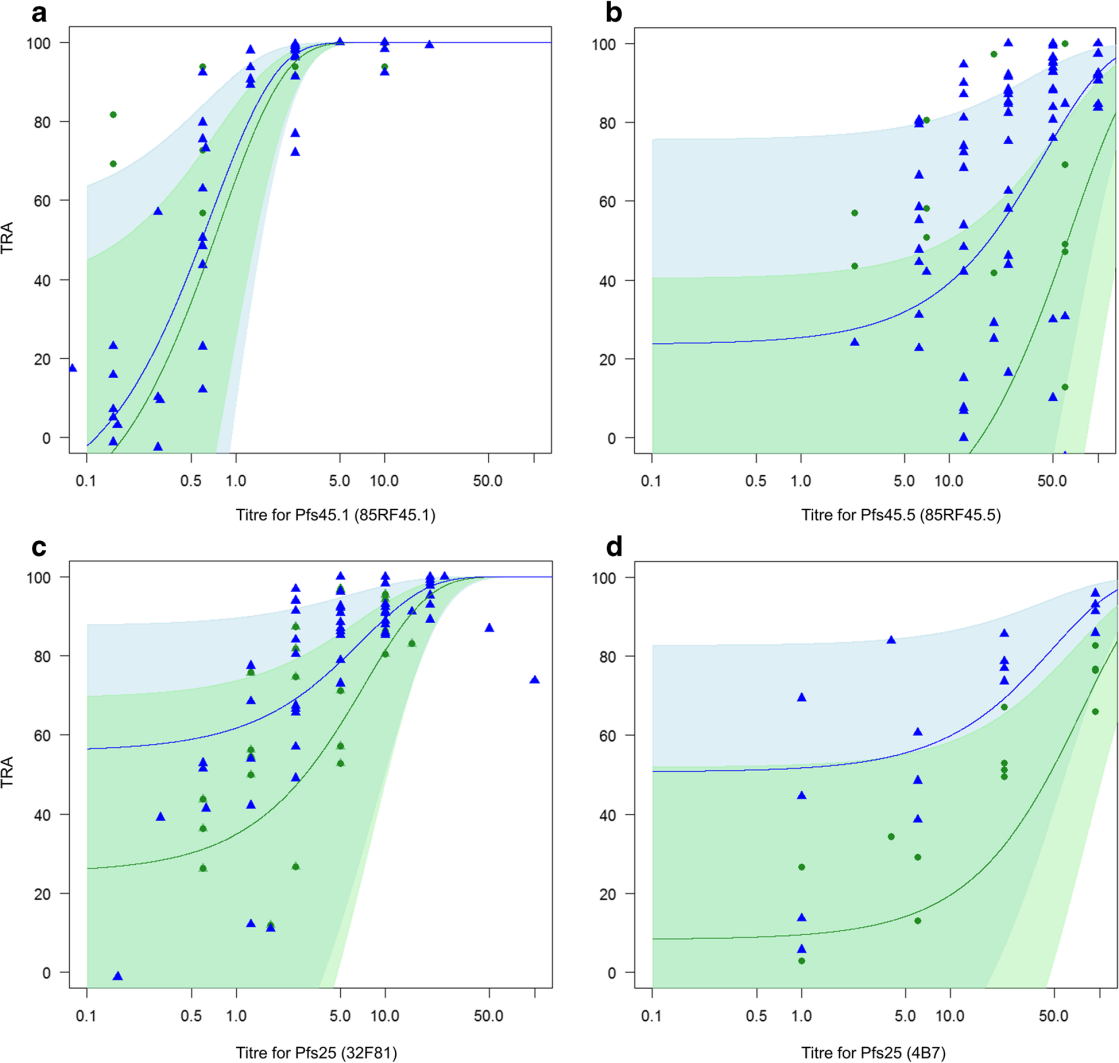
TRA of antibodies against Pfs48/45 (mAb 85RF45.1 and mAb 85RF45.5) and Pfs25 (mAb 32F81 and mAb 4B7) in *An. gambiae* and *An. stephensi* mosquitoes. Figures show the estimates of TRA as relative reduction in oocyst intensity for each experiment (dots for *An. gambiae* experiments, triangles for *An. stephensi* experiments) and the GLMM model predictions and confidence intervals (lines and shaded areas). TRA of mAb in *An. gambiae* is shown in *green* and for *An. stephensi* it is shown in *blue*. **a** Relation between *An. gambiae* and *An. stephensi* for TRA of mAb 85RF45.1. **b** Relation between *An. gambiae* and *An. stephensi* for TRA of mAb 85RF45.5. **c** Relation between *An. gambiae* and *An. stephensi* for TRA of mAb 32F81. **d** Relation between *An. gambiae* and *An. stephensi* for TRA of mAb 4B7. All TRA calculations were made using human serum controls

The highly potent mAb 85RF45.1 showed high levels of TRA in both mosquito species. At a concentration of 10 μg/ml, the number of oocysts was reduced by 99.8% (95% CI: 99.5–99.9%) and 99.9% (95% CI: 99.56–99.98%) in *An. stephensi* and *An. gambiae*, respectively (Fig. 3a). At a concentration of 2.5 μg/ml near complete blockade was also observed for both species [*An. stephensi*: 96.2% (95% CI: 94.3–97.4); *An. gambiae*: 96% (95% CI: 93.5–97.5)] while more variable levels of TRA were observed at lower concentrations. For mAb 85RF45.1, there was no significant effect of mosquito species on TRA and on the association between mAb concentration and TRA (*z*_(2133)_ = -0.45, *P* = 0.65 and *z*_(2133)_ = -1.66, *P* = 0.10, respectively).

mAb 85RF45.5 is a much less potent mAb against Pfs48/45 [29] and at none of the tested concentrations was TRA consistently above the arbitrary threshold of 80% for reproducible SMFA experiments [39]. TRA estimates for 85RF45.5 were similar between *An. stephensi* and *An. gambiae* mosquitoes (Fig. 3b) and there was no significant effect of mosquito species on TRA and on the association between mAb concentration and TRA (*z*_(2969)_ = 0.87, *P* = 0.38 and *z*_(2969)_ = -0.59, *P* = 0.55, respectively).

For mAb 32F81 against Pfs25 we found high TRA activity in both mosquitoes species at concentrations of 15 μg/ml [*An. stephensi*: 91.2% (95% CI: 87.3–93.8) TRA; *An. gambiae*: 82.8% (95% CI: 66.6–91.1)] and 10 μg/ml [*An. stephensi*: 89.6% (95% CI: 80.1–94.4); *An. gambiae:* 82.5% (95% CI: 6.0–96.8) TRA]. Below 10 μg/ml, TRA activity remained relatively high in *An. stephensi* [5 μg/ml: 85.8% (95% CI: 74.0–92.2); 2.5 μg/ml: 76.3% (95% CI: 50.4–88.7)] but dropped below statistically significant levels in *An. gambiae* (Fig. 3c). The same pattern was observed for antibody mAb 4B7 against Pfs25: we found high TRA in both mosquitoes species at concentrations of 94 μg/ml [*An. stephensi*: 92% (95% CI: 86.4–95.3), *An. gambiae*: 67% (95% CI: 52.8–77.0)]. Below this concentration, TRA remained relatively high in *An. stephensi* [23.5 μg/ml: 78.4% (95% CI: 67.7–85.6); 5.9 μg/ml: 49.6% (95% CI: 25.3–66.0)] but dropped in *An. gambiae* [23.5 μg/ml: 44.7% (95% CI: 16.1–63.6%); 5.9 μg/ml: below significant levels] (Fig. 3d). *Anopheles stephensi* supported a significantly higher TRA than *An. gambiae* for both anti-Pfs25 mAb (32F81, *z*_(2594)_ = -3.95, *P* < 0.0001; 4B7, *z*_(1070)_ = -4.68,*P* < 0.0001). This difference was significantly reduced by increasing antibody concentration in 4B7 (*z*_(1070)_ = -4.21, *P* < 0.0001).

Independent experiments were conducted at the Laboratory of Malaria and Vector Research (LMVR) using a very similar SMFA protocol [17] and provide data that support the above findings. In those experiments three mouse monoclonal antibodies (3E12, anti-Pfs48/45 mAb; 1B3, anti-Pfs230 mAb; 4B7, anti-Pfs25 mAb) were tested by SMFA; no difference was observed in TRA between the two mosquito species for anti-Pfs48/45 and anti-Pfs230 antibodies whilst TRA estimates were higher in *An. stephensi* for anti-Pfs25 mAb (Additional file 5: Figure S4).

### Assessments of transmission-reducing activity of human serum IgG

**Fig. 4:**
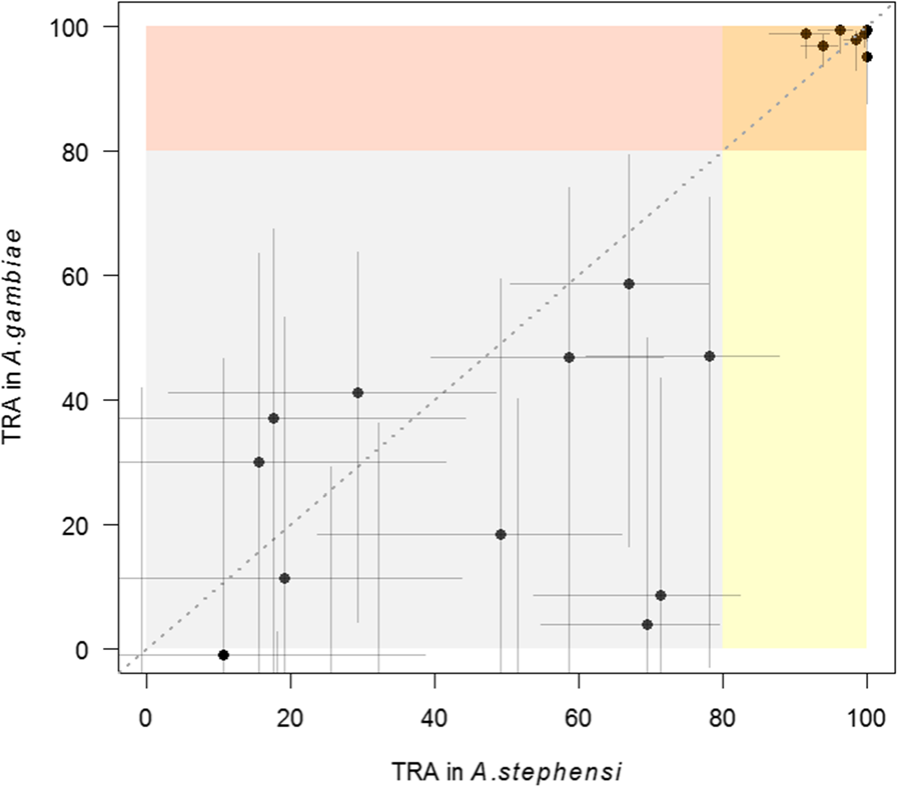
TRA of human serum IgG from Cameroon and Burkina Faso and serum IgG from Dutch expatriate donor SP in *An. gambiae* and *An. stephensi* mosquitoes. All TRA calculations were made using FCS controls. TRA calculated using mean oocyst intensity assessments from oocyst intensity data from feeds with human serum IgG. R was calculated based on deviation from a perfect linear association (x = y)

Twenty-six human serum samples were selected and tested in direct paired experiments with the same parasite material and feeders from the same feeder chain used for *An. stephensi* and *An. gambiae*. Four of 5 samples from expatriates showed ≥ 99% TRA; the other sample showing negligible activity in the SMFA. Serum from one gametocyte carrier from Cameroon and 4 from Burkina Faso showed high and reproducible levels of TRA (84–100%). All other field samples showed intermediate to low levels of TRA (Additional file 6: Table S2). There was a very strong association between SMFA estimates using *An. stephensi* and *An. gambiae* (Spearman's correlation coefficient *r* = 0.85, 95% CI: 0.71–0.93, *P* < 0.0001; Fig. 4).

Overall, TRA was significantly higher in *An. stephensi* than in *An. gambiae* mosquitoes (as expressed by the interaction term between species and intervention, GLMM *P* < 0.01) but with an average difference of only 12.8% (95% CI:16.2–19.9%). As was seen in the previous analyses the greatest difference was observed at lower TRA estimates, with sera giving > 80% TRA being highly comparable in this experiment (Fig. 4). TBA estimates also showed a good correlation, and no significant difference, between *An. stephensi* and *An. gambiae* (Additional file 4: Figure S3).

## Discussion

We compared TRA estimates of antibodies obtained in the SMFA using the commonly used *An. stephensi* and the important African malaria vector *An. gambiae*. There was a strong correlation in TRA efficacy estimates generated using the two mosquito species. For pre-fertilization anti-Pfs48/45 monoclonal antibodies and for the highest concentrations of post-fertilization anti-Pfs25 antibodies tested here, we found no differences in absolute TRA estimates between the mosquito species. When partially effective concentrations of antibodies against Pfs25 were used, we observed higher TRA estimates for *An. stephensi*.

*Anopheles stephensi* is commonly used as mosquito vector in the SMFA as well-established colonies are highly permissive and allow high oocyst and sporozoite densities to be achieved [17, 28]. Several characteristics of *An. stephensi* make this species markedly different from vectors that play a prominent role in the natural transmission of *P. falciparum* in Africa. *An. gambiae* has a potent thioester-containing protein-1 (TEP1) dependent immune system that is effective against *Plasmodium* infection. In our *An. gambiae* colony silencing TEP1 results in markedly higher infection rates with several *P. falciparum* strains [40]. The TEP1 response is absent or at least markedly lower in *An. stephensi* [24, 41]. The difference in the interaction between *Plasmodium* and different anophelines is illustrated by the *Plasmodium Pfs47* gene that is essential to avoid TEP-1 mediated killing in *An. gambiae* but plays no evident role in sporogonic development in *An. stephensi* [18, 41]. In addition, expression profiles of proteolytic enzymes during digestion of the blood meal vary between *An. stephensi* and *An. gambiae* mosquitoes [42, 43], which may affect parasite survival and infectivity. The composition of bacterial midgut flora can also vary between mosquito species and may further influence the efficiency of *Plasmodium* transmission [44–48] although this may be less important in our mosquito colonies that were both maintained in the same laboratory.

Despite these differences, our findings provide no evidence for considerable differences between *An. stephensi* and *An. gambiae* in assessing TRA for pre-fertilization antibodies, reflected by indistinguishable TRA estimates for mAb against Pfs48/45 and highly similar TRA estimates for IgG from sera of naturally exposed individuals where antibodies are exclusively expected against pre-fertilization antigens [9]. Similarly, the highest concentrations of mAb against the post-fertilization antigen Pfs25 used in our experiments, effectively reduce transmission in both *An. stephensi* and *An. gambiae*. Our findings are less conclusive for lower, partially effective, concentrations of two different sources of anti-Pfs25 mAb where TRA estimates appeared significantly higher for *An. stephensi*. Pfs25 is translationally repressed in gametocytes and expressed in zygote and ookinete stages where it facilitates midgut invasion [49, 50]. A previous study reported that Pfs25 specific IgG antibodies from immunized mice had comparable TRA efficacy in *An. gambiae* and *An. stephensi* mosquitoes based on median number of oocysts but, similar to our findings, the lowest IgG concentration resulted in higher infection rates in *An. gambiae*. [51]. Whilst independent experiments in a different laboratory confirmed statistically significant higher estimate of TRA in *An. stephensi* compared to *An. gambiae* for the post-fertilization antibody Pfs25 4B7 (Additional file 5: Figure S4), further work should elucidate whether this difference between mosquito species is also apparent for other post-fertilization antibodies and whether this reflects differences in antibody effectiveness that may need to be sustained for > 20 h in the mosquito gut to prevent further sporogonic development after ookinetes have formed [13, 52].

We designed our experiments to test TRA using reductions in oocyst density as the primary outcome measure. Ultimately, oocyst prevalence or the proportion of infected mosquitoes is a more relevant outcome to predict the community impact of interventions [5], an impact that may only become apparent after multiple transmission cycles [53]. Reductions in oocyst intensity (TRA) are typically less dependent on the efficiency of transmission and are strongly associated with reductions in oocyst prevalence (TBA) [37]. Our approach thus describes a suitable first step in candidate prioritization where multiple concentrations of transmission-blocking compounds are tested repeatedly using informative oocyst exposure in a highly permissive mosquito vector. However, compounds with activities downstream of gametocyte development might require testing in different species combinations to confirm potency in a range of epidemiologically relevant mosquito species. Our findings suggest that the SMFA with *An. stephensi* is an appropriate system for an important initial screen for TRA of transmission-blocking compounds. Ultimately, lead candidates will require testing at a range of oocyst intensities [5, 37] and ideally against multiple parasite strains. Testing candidates in feeding experiments with lower parasite exposure than routinely used in the SMFA (i.e. lower oocyst prevalence and intensity in control mosquitoes) will be of particular relevance for partially effective interventions that may still have a considerable impact on transmission [53]. Since the SMFA will always fall short of the natural situation of malaria transmission, testing interventions in the direct membrane feeding assays (DMFA) using gametocyte carriers with naturally circulating gametocyte isolates at natural densities and local mosquito species may provide additional information [54] that will help bridge the gap between in vitro candidate prioritization and predicting the efficacy of candidate interventions in real life.

## Conclusions

Our study shows that monoclonal antibodies of target vaccines have comparable levels of transmission reduction in *Anopheles stephensi* and *Anopheles gambiae* mosquitoes. In addition, human serum IgG sample also have comparable levels of transmission reduction in *An. stephensi* and *An. gambiae* mosquitoes. These finding support the use of *An. stephensi* in the SMFA for target prioritization. However, compounds with activities downstream of gametocyte development may require additional testing in different species combinations to confirm potency in a range of epidemiologically relevant mosquito species.

## Additional files

Additional file 1: Table S1. TRA and TBA estimates of human serum IgG samples. IgG concentration is shown in mg/ml. TRA estimates are shown as % reduction in oocyst intensity with 95% CI and TBA estimates are shown as % reduction in oocyst prevalence with 95% CI. (XLSX 31 kb)

Additional file 2: Figure S1. The ranking of estimates of TBA of antibodies against Pfs48/45 (mAb 85RF45.1 and mAb 85RF45.5) and Pfs25 (mAb 32F81 and mAb 4B7) in *An. gambiae* and *An. stephensi* mosquitoes. TBA of transmission effective mAb 85RF45.1 (blue), mAb 85RF45.5 (red), mAb 32F81 (green) and mAb 4B7 (black) in *An. gambiae* depending on TBA in *An. stephensi* mosquitoes. Dots and triangles represent the predicted TBA, while lines represent 95% confidence intervals in *An. gambiae* and *An. stephensi. (TIFF 1302 kb)*

Additional file 3: Figure S2. TBA of antibodies against Pfs48/45 (mAb 85RF45.1 and mAb 85RF45.5) and Pfs25 (mAb 32F81 and mAb 4B7) in *An. gambiae* and *An. stephensi* mosquitoes. Figures show the estimates of TBA as relative reduction in oocyst prevalence for each experiment (dots for *An. gambiae* experiments, triangles for *An. stephensi* experiments) and the GLMM model predictions and confidence intervals (lines and shaded areas). TBA of mAb in *An. gambiae* is shown in green and for *An. stephensi* it is shown in blue. **A** Relation between *An. gambiae* and *An. stephensi* for TBA of mAb 85RF45.1 **B** Relation between *An. gambiae* and *An. stephensi* for TBA of mAb 85RF45.5 **C** Relation between *An. gambiae* and *An. stephensi* for TBA of mAb 32F81. **D** Relation between *An. gambiae* and *An. stephensi* for TBA of mAb 4B7. All TRA calculations were made using human serum controls. (TIFF 4772 kb)

Additional file 4: Figure S3. Correlation between ranking of TBA of human IgG samples in *An. stephensi* and *An. gambiae.* TBA of human serum IgG in *An. gambiae* depending on TBA in *An. stephensi* mosquitoes. Dots represent the predicted TBA, while lines represent 95% confidence intervals in *An. gambiae* and *An. stephensi. (TIFF 1082 kb)*

Additional file 5: Figure S4. Outcomes of independently conducted experiments with mouse monoclonal antibodies against Pfs48/45 (mAb 3E12), Pfs230 (mAb 1B3) and Pfs25 (mAb 4B7). Presented are estimates of transmission reducing activity in *An. stephensi* (blue) and *An. gambiae* (red). Box plots indicate median TRA with quartiles and range. The n indicates the number of experiments. *P-*values are for the comparisons between mosquito species. (TIFF 1206 kb)

Additional file 6: Table S2. Overview of excluded SMFA experiments. Controls in experiments where prevalence was < 70% are highlighted in red. (TIFF 262 kb)

## Abbreviations

CI: Confidence interval
FCS: Foetal calf sera
GLMMS: Generalized linear mixed models
LMVR: Laboratory of Malaria and Vector Research
mAb: Monoclonal antibody
SMFA: Standard membrane feeding assay
SSM-VIMT: Sexual-sporogonic-mosquito vaccines that interrupt malaria transmission
TBA: Transmission-blocking activity
TEP1: Thioester-containing protein-1
TRA: Transmission-reducing activity
VIMT: Vaccines that interrupt malaria transmission
