# Supplementary figures and images for: Comparative assessment of An. gambiae and An. stephensi mosquitoes to determine transmission-reducing activity of antibodies against P. falciparum sexual stage antigens
Source: Parasit Vectors. 2017 Oct 17;10:489. doi: 10.1186/s13071-017-2414-z (PMC5646129; doi:10.1186/s13071-017-2414-z)

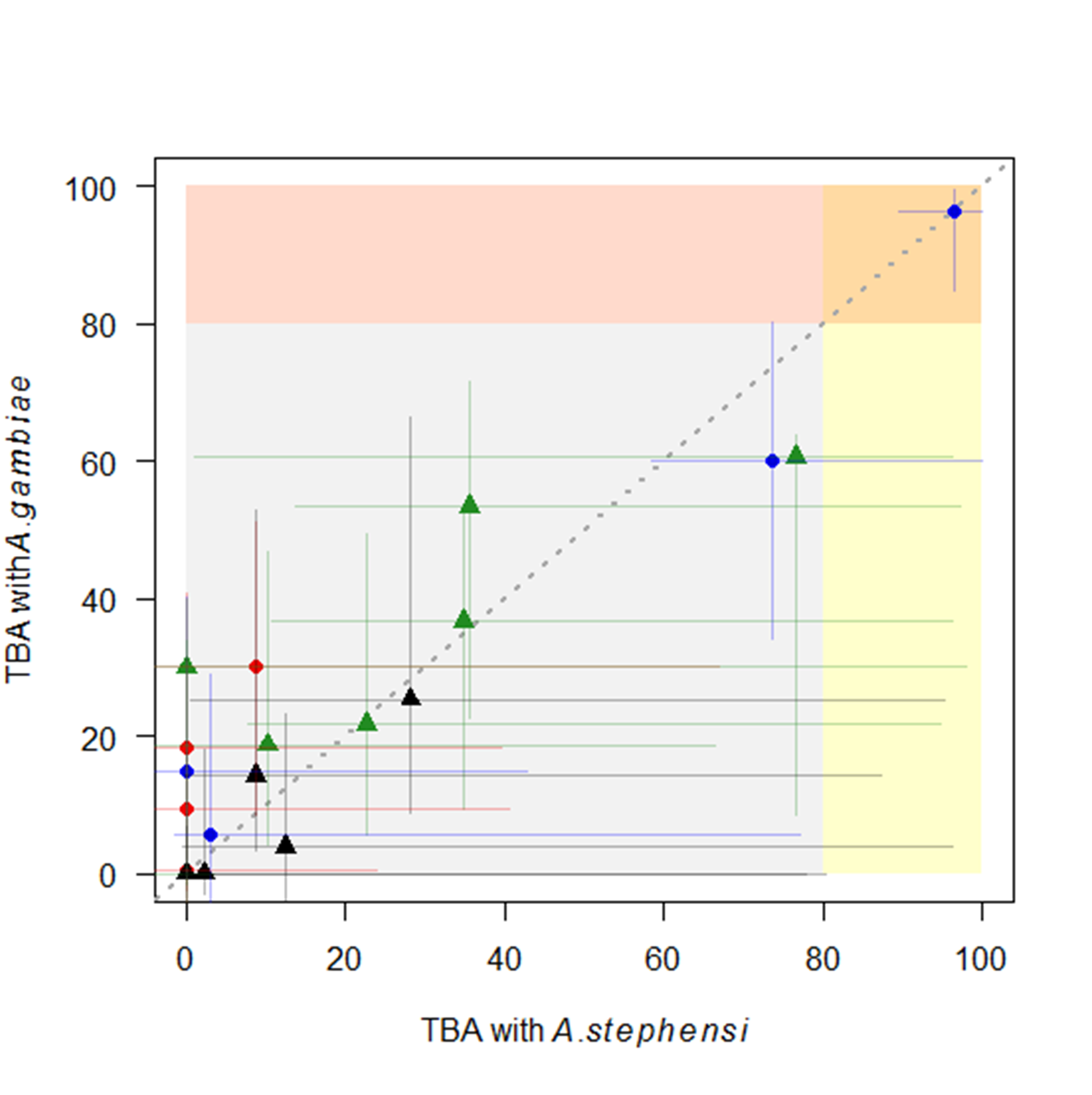

Supplement: Supplementary file 2 — The ranking of estimates of TBA of antibodies against Pfs48/45 (mAb 85RF45.1 and mAb 85RF45.5) and Pfs25 (mAb 32F81 and mAb 4B7) in An. gambiae and An. stephensi mosquitoes. TBA of transmission effective mAb 85RF45.1 (blue), mAb 85RF45.5 (red), mAb 32F81 (green) and mAb 4B7 (black) in An. gambiae depending on TBA in An. stephensi mosquitoes. Dots and triangles represent the predicted TBA, while lines represent 95% confidence intervals in An. gambiae and An. stephensi. (TIFF 1302 kb) [file 13071_2017_2414_MOESM2_ESM.tif]

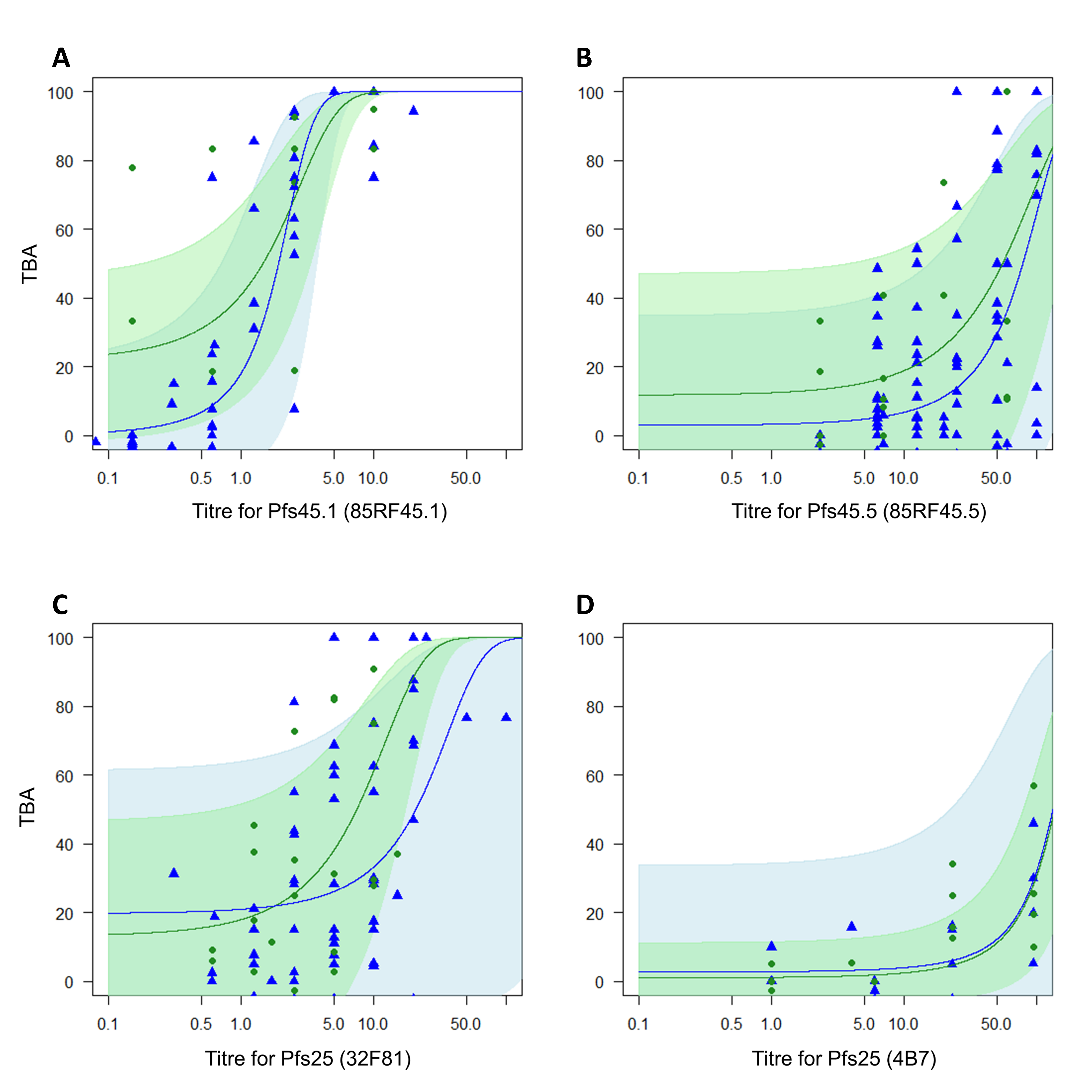

Supplement: Supplementary file 3 — TBA of antibodies against Pfs48/45 (mAb 85RF45.1 and mAb 85RF45.5) and Pfs25 (mAb 32F81 and mAb 4B7) in An. gambiae and An. stephensi mosquitoes. Figures show the estimates of TBA as relative reduction in oocyst prevalence for each experiment (dots for An. gambiae experiments, triangles for An. stephensi experiments) and the GLMM model predictions and confidence intervals (lines and shaded areas). TBA of mAb in An. gambiae is shown in green and for An. stephensi it is shown in blue. A Relation between An. gambiae and An. stephensi for TBA of mAb 85RF45.1 B Relation between An. gambiae and An. stephensi for TBA of mAb 85RF45.5 C Relation between An. gambiae and An. stephensi for TBA of mAb 32F81. D Relation between An. gambiae and An. stephensi for TBA of mAb 4B7. All TRA calculations were made using human serum controls. (TIFF 4772 kb) [file 13071_2017_2414_MOESM3_ESM.tif]

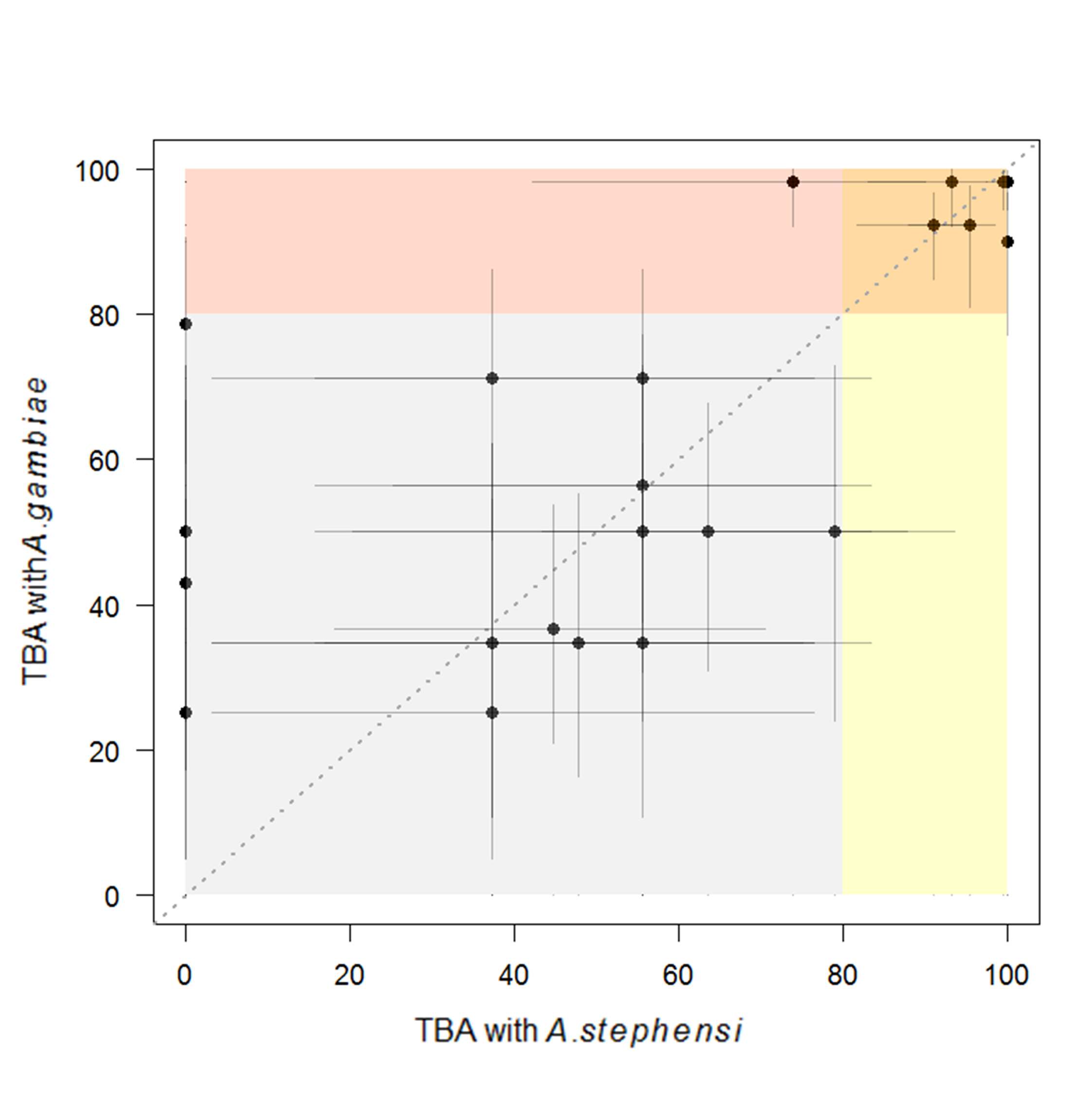

Supplement: Supplementary file 4 — Correlation between ranking of TBA of human IgG samples in An. stephensi and An. gambiae. TBA of human serum IgG in An. gambiae depending on TBA in An. stephensi mosquitoes. Dots represent the predicted TBA, while lines represent 95% confidence intervals in An. gambiae and An. stephensi. (TIFF 1082 kb) [file 13071_2017_2414_MOESM4_ESM.tif]

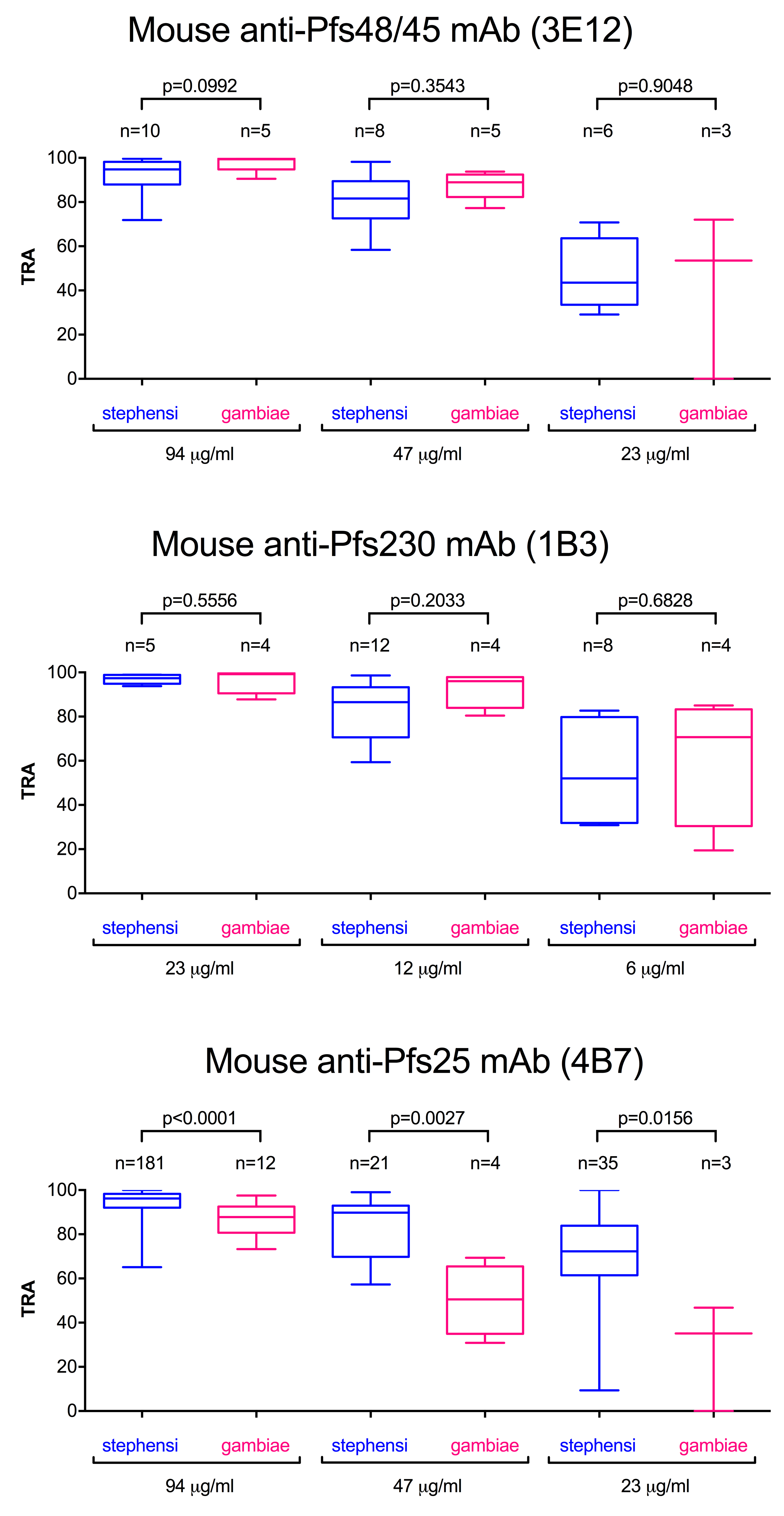

Supplement: Supplementary file 5 — Outcomes of independently conducted experiments with mouse monoclonal antibodies against Pfs48/45 (mAb 3E12), Pfs230 (mAb 1B3) and Pfs25 (mAb 4B7). Presented are estimates of transmission reducing activity in An. stephensi (blue) and An. gambiae (red). Box plots indicate median TRA with quartiles and range. The n indicates the number of experiments. P-values are for the comparisons between mosquito species. (TIFF 1206 kb) [file 13071_2017_2414_MOESM5_ESM.tiff]

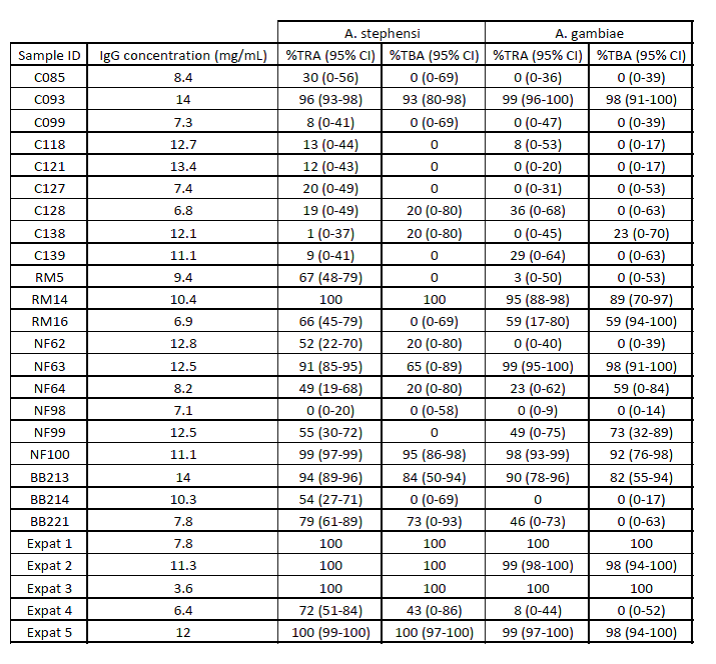

Supplement: Supplementary file 6 — Overview of excluded SMFA experiments. Controls in experiments where prevalence was < 70% are highlighted in red. (TIFF 262 kb) [file 13071_2017_2414_MOESM6_ESM.tif]
